# Dataset of pollination traits in Fabales

**DOI:** 10.1016/j.dib.2022.108480

**Published:** 2022-07-22

**Authors:** Deniz Aygören Uluer, Félix Forest, Julie A. Hawkins

**Affiliations:** a School of Biological Sciences, University of Reading, Lyle Building, Whiteknights, Reading, Berkshire RG6 6BX, United Kingdom; b Department of Plant and Animal Production, Cicekdagi Vocational College, Ahi Evran University, Cicekdagi, Kirsehir 40700, Turkiye; c Royal Botanic Gardens, Kew, Richmond, Surrey TW9 3DS, United Kingdom

**Keywords:** Flower, Leguminosae, Morphology, Pollination, Polygalaceae, Quillajaceae, Surianaceae

## Abstract

The data presented in this paper is supporting the research article “Reconstructing an historical pollination syndrome: keel flowers” (Aygören Uluer et al., 2022). We present a dataset containing information on number of species, geographic distribution, floral type (keeled or not), presence or absence of fused petals, floral symmetry, presence or absence of a pentamerous corolla (petals+petaloid sepals in Polygalaceae), androecium type, presence or absence of enclosed reproductive organs, presence or absence of three distinct petal types (petals+ petaloid sepals in Polygalaceae), flower size, corolla size (i.e., in open flower) and/or filament size (i.e., entire filament size particularly in subfamily Caesalpinioideae), flower colour, UV reflectance, habit, height, inflorescence type and inflorescence size for 758 Fabales genera. The information was obtained from hundreds of appropriate, previously published sources. This the largest morphological dataset constructed for Fabales to date, and the data presented in this article can be used for morphology, biogeography, ancestral state, ancestral area analyses of any Fabales clades.

## Specifications Table

**Table:** 

Subject	Biology/ Plant Science
Specific subject area	Plant morphology, biogeography
Type of data	Table
How the data were acquired	An extensive literature review was conducted.
Data format	Filtered
Description of data collection	Related information was obtained from every appropriate, previously published source, such as, articles, flora books, web pages.
Data source location	University of Reading library (Reading, UK)Kew Gardens library and herbarium (London, UK) • Various studies and webpages.
Data accessibility	Repository name: Mendeley Data Repository Data identification number: doi.org/10.17632/hh42swfh9w.3 Direct URL to data: https://data.mendeley.com/datasets/hh42swfh9w/3
Related research article	D. Uluer Aygören, F. Forest, S. Armbruster, J. A. Hawkins, Reconstructing an historical pollination syndrome: keel flowers. BMC Ecol Evo 22, 45 (2022). https://doi.org/10.1186/s12862–022–02003-y

## Value of the Data

• Many morphological traits, such as, corolla symmetry, flower size, flower colour and height of flowers from ground contribute to pollinator attraction [2], [3], [4], [5], [6], [7]. However, a detailed investigation for these important morphological traits for Fabales has never been conducted. This is the first and the largest dataset to date, for 15 morphological characters, that are important for Fabales pollination, as well as geographic information and number of species for 758 Fabales genera.
• The dataset benefits researchers interested in any morphology, biogeography, ancestral state and/or ancestral area analyses of any Fabales clades.
• The dataset can be used for investigation of disparification of keel flowers in Leguminosae and in Polygalaceae, may contribute to the work of the Legume Phylogeny Working Group (LeMorWoGru), and could also be expanded in the future to cover external links, maps and figures.
• The dataset we present contains information of the 15 morphological traits were selected as potentially the most important from the point of view of a pollinator: floral type (keeled or not), presence or absence of fused petals, floral symmetry, presence or absence of a pentamerous corolla (petals+ petaloid sepals in Polygalaceae), androecium type, presence or absence of enclosed reproductive organs, presence or absence of three distinct petal types (petals+ petaloid sepals in Polygalaceae), flower size, corolla size (i.e., in open flower) and/or filament size(i.e., entire filament size particularly in subfamily Caesalpinioideae), flower colour, UV reflectance (e.g., FReD: the floral reflectance database) [8], habit, height, inflorescence type and inflorescence size.
• Some parts of the data presented in this paper was used for analyses in Aygören Uluer et al. [1]. However, other parts, such as, flower colour, UV reflectance, number of species, corolla/filament size are newly added.

## 1 Data Description

All available information for 758 Fabales genera and their species is organized in an Excel sheet including number of species, geographic distribution, floral type (keeled or not), presence or absence of fused petals, floral symmetry, presence or absence of a pentamerous corolla (petals+ petaloid sepals in Polygalaceae), androecium type, presence or absence of enclosed reproductive organs, presence or absence of three distinct petal types (petals+ petaloid sepals in Polygalaceae), flower size, corolla size (i.e., in open flower) and/or filament size(i.e., entire filament size particularly in subfamily Caesalpinioideae), flower colour, UV reflectance, habit, height, inflorescence type and inflorescence size. Very rarely calyx, wing and filament (stamen) lengths were also provided in the petals/ corolla/ keel/ filament (stamen) size column (here keel size is probably more important for a pollinator, however all available information was added to this column as a future reference for some taxa). Table 1 shows the completeness of the current dataset.

**Table 1:** Completeness of the current dataset.

	Leguminosae	Polygalaceae	Surianaceae	Quillajaceae
# of genera included	732 (96%)	20 (74%)	5 (100%)	1 (100%)
Geography	100%	100%	100%	100%
Flower type	100%	100%	100%	100%
Fused petals or not	62%	95%	40%	100%
Corolla symmetry	98%	100%	100%	100%
Pentamerous corolla	94%	95%	100%	100%
Androecium type	89%	100%	100%	100%
Enclosed reproductive organs	95%	85%	100%	100%
Three distinct petal/sepal	99%	100%	100%	100%
Flower size	91%	85%	100%	100%
Corolla/filament size	60%	75%	60%	0%
Flower colour	94%	100%	100%	100%
UV reflectance	4%	5%	0%	0%
Habit	98%	100%	100%	100%
Height	89%	80%	100%	100%
Inflorescence type	95%	95%	80%	100%
Inflorescence size and/or flower number	65%	90%	40%	0%

Both Lewis [9] and LPWG [10] used as the taxonomic backbone. In this file, "/" and ";" represent different sources or information about different species. “?” represents information should be accepted with caution. Empty cells represent unavailable information. "Refer to FReD web-page" was used to emphasize the large amount of information that at http://www.reflectance.co.uk/. NA: not applicable. K: keeled, N: non-keeled, P: pseudo-papilionoid. Monodelp: monodelphous, diadelp: diadelphous. S: South, W: West, C: Central, N: North, SE: South-East, SC: South-Central, SW: South-West, NW: North-West, NE: North-East, WC: West-Central, EN: East-North. m: metre, dm: decimetres, cm: centimetres, mm: millimetres, ‘: foot (plural form feet), “: inc (plural form inches).

## 2 Experimental Design, Materials and Methods

The information was obtained from every appropriate, previously published source, and the list of publications used here can be found at the end of the dataset table. Further details about the experimental design, materials and methods related to data are described at Aygören Uluer et al. [1].

## Ethics Statement

No human or animal subjects were involved in data collection.

## Funding

The first author is grateful to Republic of Turkiye Ministry of National Education for funding. The funding body played no role in the sample collection.

## Supplementary Materials

Supplementary material associated with this article (i.e., Dataset of pollination traits in Fabales) can be found at https://data.mendeley.com/datasets/hh42swfh9w/3.

## CRediT authorship contribution statement

**Deniz Aygören Uluer:** Data curation, Writing – review & editing, Writing – original draft. **Félix Forest:** Methodology, Supervision. **Julie A. Hawkins:** Conceptualization, Writing – review & editing, Methodology, Supervision.

## Declaration of Competing Interest

The authors declare that they have no known competing financial interests or personal relationships that could have appeared to influence the work reported in this paper.

## Data Availability

Dataset of pollination traits in Fabales (Reference data) (Mendeley Data).
